# Accept it or forget it: mandatory digital learning and technology acceptance in higher education

**DOI:** 10.1007/s40692-022-00244-w

**Published:** 2022-10-12

**Authors:** Thomas Lehmann, Patrick Blumschein, Norbert M. Seel

**Affiliations:** 1 Faculty 12: Pedagogy and Educational Sciences, University of Bremen, Universitäts-Boulevard 11/13, 28359 Bremen, Germany; 2 Department of Educational Science, University of Education, Kunzenweg 21, 79117 Freiburg, Germany; 3 Department of Educational Science, University of Freiburg, Rempartstr. 11, 79098 Freiburg, Germany

**Keywords:** Digital learning, Emergency remote teaching, Higher education, UTAUT, Structural equation modeling

## Abstract

In early 2020, the COVID-19 pandemic led to a rapid shift to emergency remote teaching and mandatory digital learning in higher education. This study tested an extended causal model built on the Unified Theory of Acceptance and Use of Technology (UTAUT) under the restrictions on higher education stemming from the pandemic. Data collected from a survey among 485 students were analyzed using structural equation modeling. Confirmatory factor analyses were performed to examine the construct validity of the measurement model using polychoric correlations. Path analysis was used to test the causal model. The results indicate a psychologically sound baseline model with nine latent variables that affect students’ behavioral intention to accept and continue using technology for learning. However, the model is only partially in line with the proposed causal model based on UTAUT. The implications of these results are discussed in terms of technology acceptance and use in higher education, both under the restrictions leading to mandatory digital learning and in future.

## Introduction

In early 2020, the COVID-19 pandemic changed the field of higher education. Numerous surveys have since confirmed the negative effects of COVID-19 across many dimensions of higher education. Within a few days, all educational institutions had to switch to online teaching and learning. As a consequence of this unforeseen development, universities experienced a challenging transition to online teaching. For example, in June 2020, more than 2000 professors in Germany advocated the necessity of returning to face-to-face instruction in higher education in an open letter. However, not only professors and instructors faced difficulties with the rapid shift to digital teaching but also many students (Aristovnik et al., 2020; Händel et al., 2020; Traus et al., 2020). In general, the pandemic exposed shortcomings in higher education, especially regarding stakeholders’ deficient adjustment to the use of digital technology (Rashid & Yadav, 2020). To date, students have often needed to act in accordance with the motto *accept it or forget it* when confronted with the requirements of digital learning, whether it involves virtual tutoring, video conferencing, digital learning material, language apps, or specific training software.

This situation can be traced back to longstanding deficiencies in high-performance digital learning services and a lack of appropriate instructional design in the field of higher education (Bitzer et al., 2016). Previous studies (e.g., Bond et al., 2018; Persike & Friedrich, 2016) indicate that universities generally exploit only a small part of the opportunities presented by digital technologies and that both teachers and students use only a limited number of technologies for predominantly assimilative tasks. Consequently, the practice of higher education is largely characterized by conventional teaching methods—partially enriched with instructional media—and at best by blended learning scenarios. In contrast, in their everyday lives, most students are familiar with using information technologies. In a comparative study, Jefferies et al. (2016) determined that tertiary students in Germany (similarly to those in other countries) demonstrate extensive ownership and use of digital technologies to support learning, with high levels of perceived competence (see also Henderson et al., 2015), whereas they may experience a lack of opportunity regarding digital learning as part of their academic studies.

To some extent, this situation is difficult to explain because the efficient use of digital learning environments in higher education has advanced in recent years to become an important research topic from both a scientific and a practical perspective. Researchers from diverse disciplines have attempted to identify the factors leading to successful learning with digital media in higher education (e.g., Kümmel et al., 2020; Schmidt-Borcherding et al., 2020). Many technology acceptance studies have aimed to develop a comprehensive understanding of the factors which influence the effective adoption of digital learning and teaching in higher education. Most studies have focused on the optional use of technology by stakeholders. In this context, the *Technology Acceptance Model* (TAM) formulated by Davis (1989) developed into a useful general framework for research, thus producing a multitude of investigations into the factors that influence the acceptance and use of digital technologies for learning in educational contexts (Granić & Marangunić, 2019; Lai, 2017). Despite its usefulness in research (see the meta-analyses of Ma & Liu, 2004; King & He, 2006), the TAM is also criticized for its limitations as regards “(1) not providing adequate insight into individuals’ perspectives of novel systems; (2) neglecting its indicators and directly investigating the external variables of perceived ease of use (PEOU) and perceived usefulness (PU); and (3) ignoring the relationship between usage attitude and usage intention” (Chao, 2019, p. 2). It has thus undergone several enhancements to gain a deeper understanding of the complexities of technology acceptance in education (e.g., Lee et al., 2003; Venkatesh & Bala, 2008). Today, many consider the *Unified Theory of Acceptance and Use of Technology* (UTAUT) proposed by Venkatesh et al. (2003) as a reasonable comprehensive enhancement of the TAM that can be used as a basis for further research (e.g., Chao, 2019; Huan et al., 2015).

The UTAUT integrates several previously established acceptance models, such as the theory of reasoned action, motivation theory, and the theory of planned behavior (Venkatesh, 2000). Usually, UTAUT models focus on the optional use and acceptance of digital technologies. However, due to the constraints imposed by COVID-19, the *mandatory* use of digital learning environments became the standard in higher education. Although universities worldwide have started moving back to “normal teaching,” it remains unclear whether another wave of COVID-19 will occur. This would result in another phase of social distancing and, therefore, mandatory digital learning, as was already the case in the 2021–2022 winter semester in many countries. Unfortunately, only a few studies to date have focused on mandatory digital learning in higher education (e.g., Beaunoyer et al., 2020; Carter et al., 2020; Dečman, 2015; Evans & Le Roux, 2015; Khechine & Lakhal, 2018). Hence, it remains to be determined which intrapersonal and external factors affect students’ technology acceptance and behavioral intention to use technology for mandatory distance learning. Therefore, the aim of the present study is to generate fresh insights into the central factors in students’ acceptance and use of technology in emergency remote teaching by testing a particular UTAUT model focused on mandatory digital learning in higher education under the constraints caused by the COVID-19 pandemic. The interplay between technology-related and personal aspects are investigated using this UTAUT model.

## Theoretical foundation: the UTAUT model

**Fig. 1:**
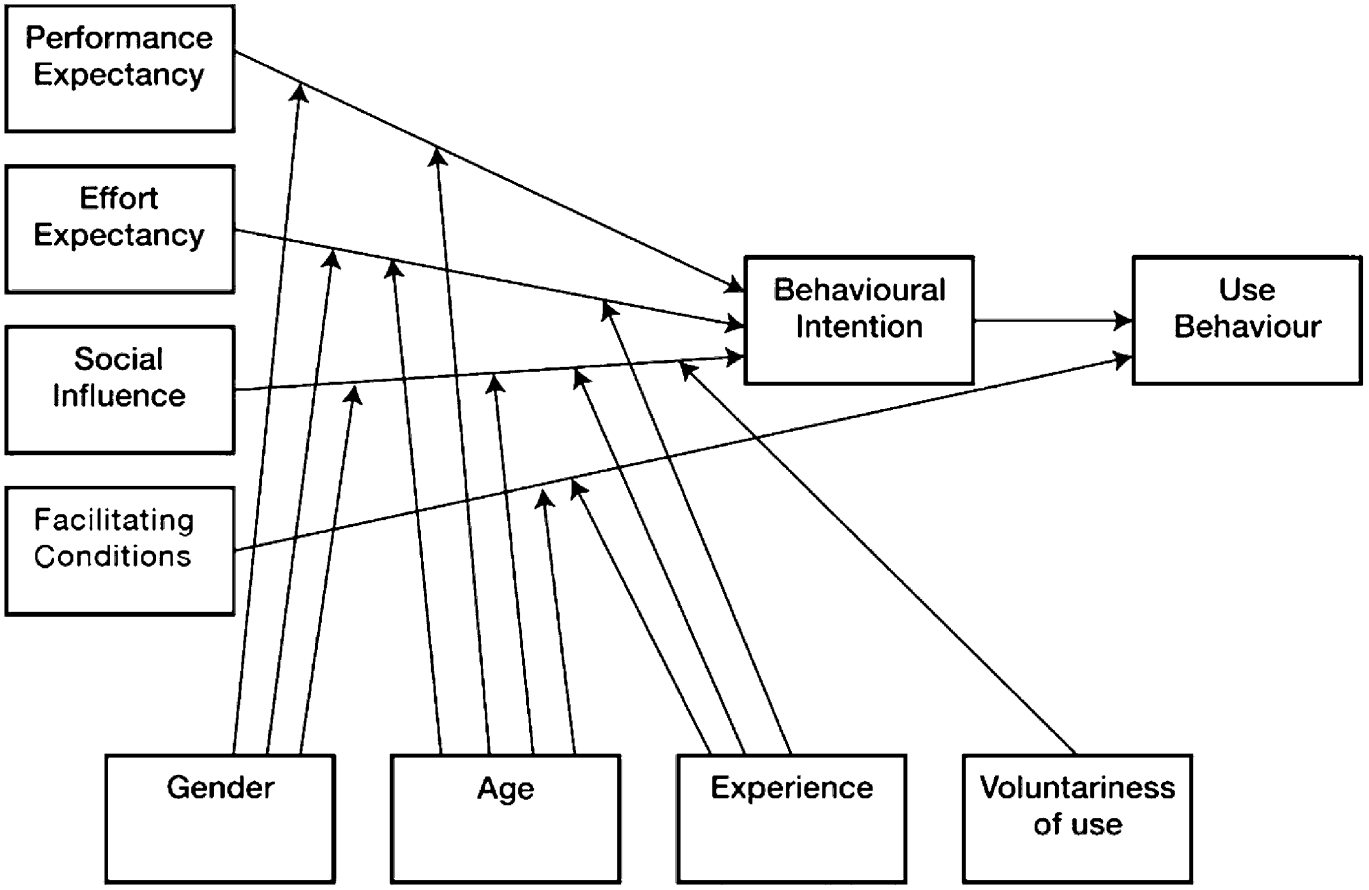
The UTAUT model (Venkatesh et al., 2003, p. 447)

The UTAUT is an elaboration of the TAM and was originally proposed by Venkatesh et al. (2003). Whereas, the TAM distinguishes between two primary factors influencing an individual’s intention to use new technology (*perceived ease of use* and *perceived usefulness*), the UTAUT identifies four key factors (*performance expectancy*, *effort expectancy*, *social influence*, and *facilitating conditions*) and four moderators (age, gender, experience, and voluntariness) related to predicting both the *behavioral intention* to use a technology and the *actual use* of technology in organizational contexts. Figure 1 shows the original UTAUT.

Due to its ability to integrate different approaches to technology acceptance, the UTAUT contributes substantially to the exploration of technology acceptance and use in the digital age. Since its introduction, many researchers have applied, integrated, and extended the model across a variety of settings, users, organizations, tasks, and locations. The UTAUT initiated an abundance of empirical research (see Venkatesh et al., 2016 for an overview), with numerous studies in the field of higher education and a particular focus on the acceptance of mobile/digital learning (e.g., Chao, 2019; Huan et al., 2015; Khechine et al., 2016; Salloum & Shaalan, 2019). Furthermore, the originating article has been cited by a large number of studies, but Williams et al. (2015) stated that of 450 citations, only 43 actually utilized the theory in empirical research. It seems that the “UTAUT’s well-defined parts, the well-accepted importance and boundary of research […] not only lead to the limited number of UTAUT extensions but also hamper further significant theoretical advancement of the theory” (Venkatesh et al., 2016, p. 338).

### The present study

In accordance with recommendations by Venkatesh et al. (2016), the present study conceptualizes individual technology acceptance at the feature level and uses UTAUT as the baseline model to refine the conceptualization and measurement of the current contextual factors in higher education. Usually, the voluntariness of use is a central component of UTAUT; thus, the actual use behavior is considered as a criterion variable (see Fig. 1). Thus, the original UTAUT model does not fit the requirements of *mandatory* digital learning as implemented in higher education under the restrictions stemming from the COVID-19 pandemic. Pragmatically, the present study adopts a particular UTAUT model proposed by Huan et al. (2015) with regard to optional mobile learning. This model has been adjusted for the specific problems associated with mandatory digital learning during emergency remote teaching. Essentially, it is suggested that successful digital learning requires students to use a variety of strategies to regulate cognitive, motivational, and behavioral aspects, as well as environmental characteristics. Thus, the key variables of the UTAUT model can be described as set out below.

First, the model includes several motivational variables. *Self-efficacy* refers to the personal belief in one’s own ability to complete tasks and reach academic goals (Bandura, 1986). *Attainment value* is related to the personal importance of performing with regard to personal values, such as achievement motivation (Eccles et al., 1983). *Performance expectancy* is defined as the extent to which an individual believes digital learning will help to attain academic gains (Venkatesh et al., 2003). Furthermore, the model includes two emotional variables. *Satisfaction* is defined as the fulfillment of subjects’ emotional expectations regarding digital learning (Chao, 2019), while *perceived enjoyment* refers to performance of or engagement in an activity due to a playful interest in that activity (Huan et al., 2015; Moon & Kim, 2001). Additionally, the metacognitive variable *self-management of learning* refers to the extent to which individuals perceive themselves as self-disciplined and engaged in autonomous learning (Huan et al., 2015; Smith et al., 2003).

These latent variables are affected by participants’ age, gender, study experience, and prior experience with digital learning, whereas the latent variables, in turn, affect the participants’ *effort expectancy*, which is defined by Huan et al. (2015) as the degree of ease associated with the actual use of the materials provided in a digital learning environment. Additionally, effort expectancy is affected by the external conditions of the learning environment, such as social influence, facilitating organizational conditions, service quality, and ubiquity (e.g., Carlsson et al., 2006; Venkatesh et al., 2003). *Social influence* is defined as the extent to which an individual perceives that important persons (e.g., the instructor or classmates) believe in the effectiveness of digital learning environments. *Facilitating conditions* refer to the availability of resources needed to engage in the learning environment. *Service (and instructional) quality* refers to the reliability, accuracy, and quality of delivered content. The *ubiquity* is considered the most important and beneficial feature of digital learning compared to traditional approaches (cf. Huan et al., 2015).

**Fig. 2:**
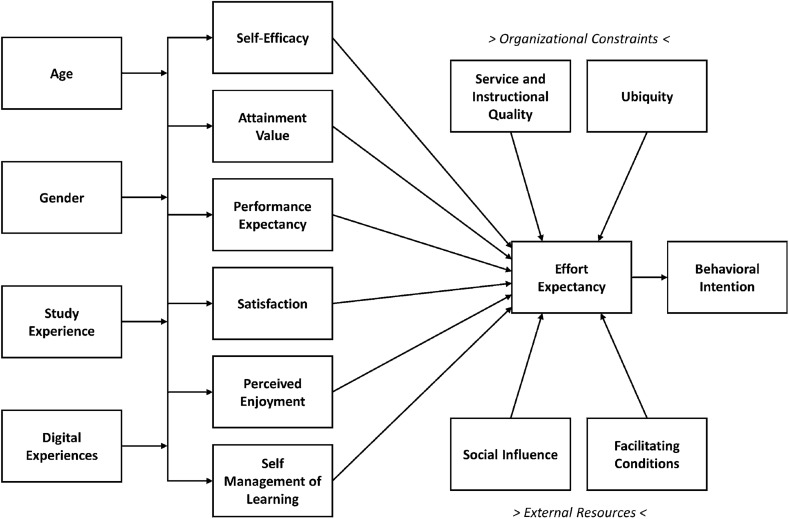
The proposed UTAUT model to be tested

To develop an increased understanding of the central factors in students’ acceptance and use of technology in emergency remote teaching and their interrelation, the current study investigates a particular UTAUT model focused on *mandatory digital learning*. Originally, the UTAUT is related to the optional use of technology (Chao, 2019; Huan et al., 2015; Venkatesh et al., 2003); thus, it presupposes the voluntariness of individuals. Accordingly, the *use behavior* and the *actual use* of digital technologies are reasonably considered as criterion variables (see Fig. 1). However, mandatory digital learning during the COVID-19 pandemic suspended the voluntariness of technology usage and, thus, the specification of *actual use* as criterion variable. In accordance with Lin et al. (2013), the *behavioral intention* of deliberately continuing to use digital learning is considered as the dependent variable in the present study. Figure 2 illustrates the a priori UTAUT model of the present study.

## Method

### Design and participants

Empirical data was collected using a cross-sectional survey design. We recruited 485 students from German universities in the North (Bremen and Hannover), the Southeast (Munich), and the Southwest (Freiburg). The students were contacted via an announcement on their university’s learning management system or through email invitations from lecturers in large courses. Participation was completely voluntary; therefore, the sample was based on convenience sampling. The students could follow a link in the announcement or the email to our online survey, which led to a webpage with information on the study and an agreement button which all subjects could click to give their informed consent for participating in the study. Next, they were asked to complete an online questionnaire based on their opinions and beliefs on accepting and using digital learning. The mean age of the sample was 25.07 years (*SD* = 5.65). A total of 76% were female, 23% were male, and 1% identified as diverse. The participants had an average study experience of 4.90 semesters (*SD* = 3.48). Altogether 62.5% were undergraduate students and 27.5% were graduate students.

### Measurement

For the instrument aimed at measuring the particular UTAUT model used in the present study, we adopted most items from the questionnaire of Huan et al. (2015) and Venkatesh et al. (2016). Additionally, items from the questionnaire used by Chao (2019) were adopted to measure participants’ *satisfaction* with digital learning. A total of 44 items were apportioned among 12 latent variables: *behavioral intention* (3 items, e.g., “I intend to engage in digital learning more often in the future [even after the pandemic].”), *effort expectancy* (4 items, e.g., “Learning how to become skilled at digital learning is easy for me.”), *self-efficacy* (4 items, e.g., “I have the knowledge and skills required for successful digital learning.”), *attainment value* (4 items, e.g., “Digital learning is helpful in achieving my learning goals.”), *performance expectancy* (4 items, e.g., “Digital learning improves my learning performance.”), *satisfaction* (4 items, e.g., “I think digital learning enhances my study effectiveness [I do things better and smarter].”), *perceived enjoyment* (4 items, e.g., “I find digital learning stimulates my curiosity.”), *self-management of learning* (3 items, e.g., “Digital learning gives me more flexibility in controlling my learning process and choosing what I want to learn.”), *social influence* (4 items, e.g., “I engage in digital learning because it is generally expected these days.”), *facilitating conditions* (3 items, e.g., “I learn digitally when there is good technical support.”), *service and instructional quality* (4 items, e.g., “It is important for digital learning material to be understandable.”), and *ubiquity* (3 items, e.g., “I would find having course materials available at any time convenient.”). Participants were instructed to indicate to what extent they agree with each item on a five-point Likert scale, from (1) “strongly disagree” to (5) “strongly agree.” Additionally, we added questions on participants’ personal data (age, sex, institution, study program, and study experience) and a single Likert-type item assessing their experience in digital learning (“I have already gained experience with digital learning in the past, even before the pandemic broke out.”) on a scale from (1) “very seldom” to (5) “very often.”

### Statistical analysis

In accordance with a common practice in UTAUT-related research, the questionnaire used in the present study applied Likert scales for categorizing subjects’ responses to items. In previous studies, Pearson correlations were used as the foundation of factor analyses for assessing the construct validity of measurement instruments with Likert scales (e.g., Aliaño et al., 2019; Chao, 2019; Huan et al., 2015; Khechine & Lakhal, 2018; Salloum & Shaalan, 2019; Tarhini et al., 2016). However, this practice is inadequate because Pearson correlations assume interval measurement scales, while Likert scales represent ordinal variables. The observable common practice of factor analyzing ordinal data is unsurprising, because textbooks (e.g., Corbetta, 2003) often condone this practice by illustrating factor analytic procedures on survey data with little or no discussion of the risks associated with using ordered-categorical (rather than interval) data. Although debate continues over the use of parametric statistical techniques for analyzing Likert scales (Carifio & Perla, 2007; Norman, 2010), empirical evidence shows that classical factor analysis based on Pearson correlations commonly can yield inaccurate results in terms of characterizing the internal structure of a scale or selecting the most informative items within each factor. *Polychoric correlations* are recommended as a more appropriate alternative for factor analyses of ordinal items. According to Asún et al. (2016), Holgado-Tello et al. (2010), and others (e.g., Flora et al., 2012; Rigdon & Ferguson, 1991), using *polychoric correlations* actually provides a more accurate assessment of the construct validity of measurement instruments incorporating Likert scales. Therefore, polychoric correlations are used in the present study for statistical analysis. The *construct validity* is assessed through a confirmatory factor analysis (CFA). The *reliability* of the instrument is evidenced by their internal consistency. In social research, the internal consistency of tests or questionnaires is estimated using Cronbach’s alpha (*α*). Although this coefficient is widely accepted, it has never been undisputed (Viladrich et al., 2017); thus, several alternatives have been provided to measure internal consistency (Peters, 2014). These coefficients typically assume that responses to the items in a survey share a single underlying construct and consequently, that their internal consistency can be derived from CFA parameter estimates. A popular reliability coefficient derived from CFA estimates is McDonald’s omega (*ω*; McDonald, 1999). The coefficient omega is based on the decomposition of the variance of a test within a factor analytic model into four parts: (1) a general factor with variance common to all measured variables, (2) a set of group factors (i.e., variance common to some of the measured variables, but not all), (3) specific factors with variance unique to each measured variable, and (4) random error (Watkins, 2017).

Similarly to most UTAUT-related studies (e.g., Chao, 2019; Salloum & Shaalan, 2019; Tarhini et al., 2016), the a priori UTAUT model (Fig. 2) is validated using the *structural equation modeling* (SEM) technique, which combines *confirmatory factor analysis* and *path analysis*. The advantage of SEM is that it considers the evaluation of the measurement model and the estimation of the structural coefficients simultaneously. The statistical analyses were conducted in the R computing environment using the *lavaan* package for CFA and SEM (Rosseel, 2012).

## Results

**Fig. 3:**
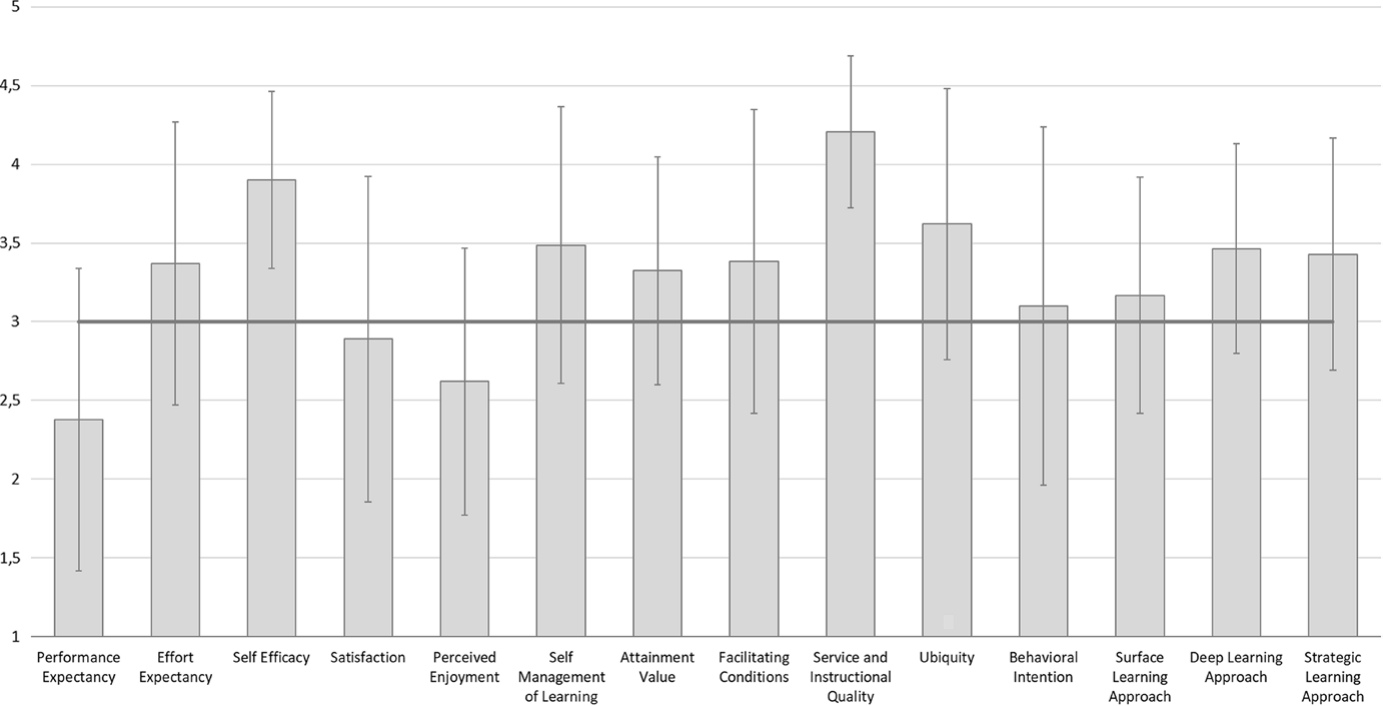
Means and standard deviations (error bars) of the subscales of the UTAUT questionnaire

In this section, we present the descriptive statistical results of the different UTAUT subscales to illustrate participants’ perception of mandatory digital learning. Figure 3 shows the means and standard deviations, with higher scores indicating more positive perceptions. A scale score of 3.0 suggests complete indecision regarding a given construct.

The scores of most UTAUT subscales exceed the undecided category of the applied Likert scales. The participants positively estimated *effort expectancy*, *self-efficacy*, *self-management of learning*, and *attainment value*. Similarly, the participants positively evaluated the contextual and organizational conditions of digital learning, as shown by the scores for the subscales *facilitating conditions*, *ubiquity*, and *service and instructional quality*. In contrast, the participants conveyed skepticism regarding *performance expectancy*, associated with reservations about *satisfaction* with and *perceived enjoyment* of the requirements of digital learning in periods of emergency remote study. Consequently, the scores of the dependent variable *behavioral intention* show the participants’ indecision concerning the request for a continuation of digital learning.

The data obtained were tested for reliability (using Cronbach’s α and McDonald’s ω to estimate internal consistency) and validity. As most of the endogenous and exogenous variables of the proposed UTAUT model have been validated in previous studies, we have assessed the construct validity of the measurement model with confirmatory factor analysis (CFA) fitted to polychoric correlations. The huge body of literature on model evaluation can roughly be classified into two categories: (a) overall model test statistics to judge whether a target model fits the data and (b) fit indices that evaluate the achievement of a model relative to a baseline model. A popular example is the *comparative fit index* (CFI) proposed by Bentler (1990), which is the standard in the R package *lavaan* for CFA. It measures the improvement in fit of a postulated target model in relation to a baseline model, which is commonly considered “the worst possible model” (Shi & Maydeu-Olivares, 2020). A rule of thumb for this index is that CFI > 0.90 indicates a good fit relative to the baseline model. Another widely used fit index is the *Tucker Lewis Index* (TLI), which penalizes overly complex models; thus, it makes the fit more conservative than CFI. Similarly to CFI, a higher TLI is better, with a reasonable fit being TLI > 0.9 (Hu & Bentler, 1999). Additionally, the *root mean square error of approximation* (RMSEA) is commonly used in CFA. The RMSEA is a “badness-of-fit measure,” with lower values indicating a better fit. According to Hu and Bentler (1999), an RMSEA ≤ 0.06 is considered acceptable, whereas an RMSEA > 0.10 indicates a poor model fit (Hoyle & Panther, 1995).

The CFA indicates that the target model with 12 factors fits the obtained data well with respect to several fit indices: CFI = 0.993, TLI = 0.993, and RMSEA = 0.051. However, several factors indicate an insufficient internal consistency. The factor loadings and reliability coefficients are summarized in Table 1.

**Table 1:** Factor loadings and reliability coefficients of the UTAUT questionnaire

Construct	No. of items	Factor loadings	Reliability coefficients	
			Omega	Alpha
Behavioral intention	3	0.777–0.946	0.652	0.640
Perceived enjoyment	4	0.622–0.784	0.769	0.768
Effort expectancy	4	0.632–0.909	0.830	0.828
Social influence	4	0.130–0.700	0.266	− 0.132
Facilitating conditions	3	0.289–0.917	0.711	0.613
Service and instructional quality	4	0.013–0.625	0.530	0.500
Self-efficacy	4	0.208–0.578	0.429	0.423
Performance expectancy	4	0.594–0.935	0.887	0.872
Satisfaction	4	0.671–0.919	0.898	0.887
Self-management of learning	3	0.549–0.867	0.666	0.653
Attainment value	4	0.003–0.846	0.489	0.445
Ubiquity	3	0.583–0.737	0.652	0.640

The reliability of the UTAUT subscales was estimated by internal consistency based on McDonald’s omega and Cronbach’s alpha. A commonly used threshold value for internal consistency is 0.70 (Lattin et al., 2003). Only five of the 12 UTAUT subscales (constructs) indicated a sufficient internal consistency with *ω* > 0.7. Given that the small number of items per scale makes high internal consistency less likely, *ω*- and *α*-coefficients > 0.6 might be considered acceptable (i.e., eight subscales provide sufficient reliability). A clear outlier is *social influence*, with *ω* = 0.266 and *α* = − 0.132. This subscale is also characterized by negative factor loadings. With the exception of the constructs *service and instructional quality* and *self-efficacy*, the factor loadings are satisfying.

Structural equation modeling and maximum likelihood estimation were applied to assess the relationships among the latent variables of the proposed UTAUT model. Accordingly, a *path analysis* was performed to test the effects of specified endogenous and exogenous variables on the criterion variable. Table 2 summarizes the maximum likelihood estimates of regression coefficients, standard error, and *p*-values.

**Table 2:** Maximum likelihood estimates of regression coefficients from the path analysis

Path			Estimate	SE
Facilitating conditions	←	Service and instructional quality	0.590***	0.092
Performance expectancy	←	Effort expectancy	0.313***	0.037
	←	Facilitating conditions	0.514***	0.034
Effort expectancy	←	Digital experience	0.065*	0.034
Satisfaction	←	Effort expectancy	0.211***	0.030
	←	Performance expectancy	0.491***	0.036
	←	Facilitating conditions	0.391***	0.033
	←	Service and instructional quality	− 0.001	0.055
Self-efficacy	←	Age	− 0.008*	0.004
	←	Study experience	0.002	0.006
	←	Effort expectancy	0.327***	0.024
	←	Performance expectancy	0.072**	0.024
Behavioral intention	←	Self-efficacy	− 0.131*	0.052
	←	Performance expectancy	0.156***	0.045
	←	Satisfaction	0.584***	0.048
	←	Facilitating conditions	0.373***	0.038
	←	Service and instructional quality	0.000	0.057

*p < .05; **p < .01; ***p < .001

**Fig. 4:**
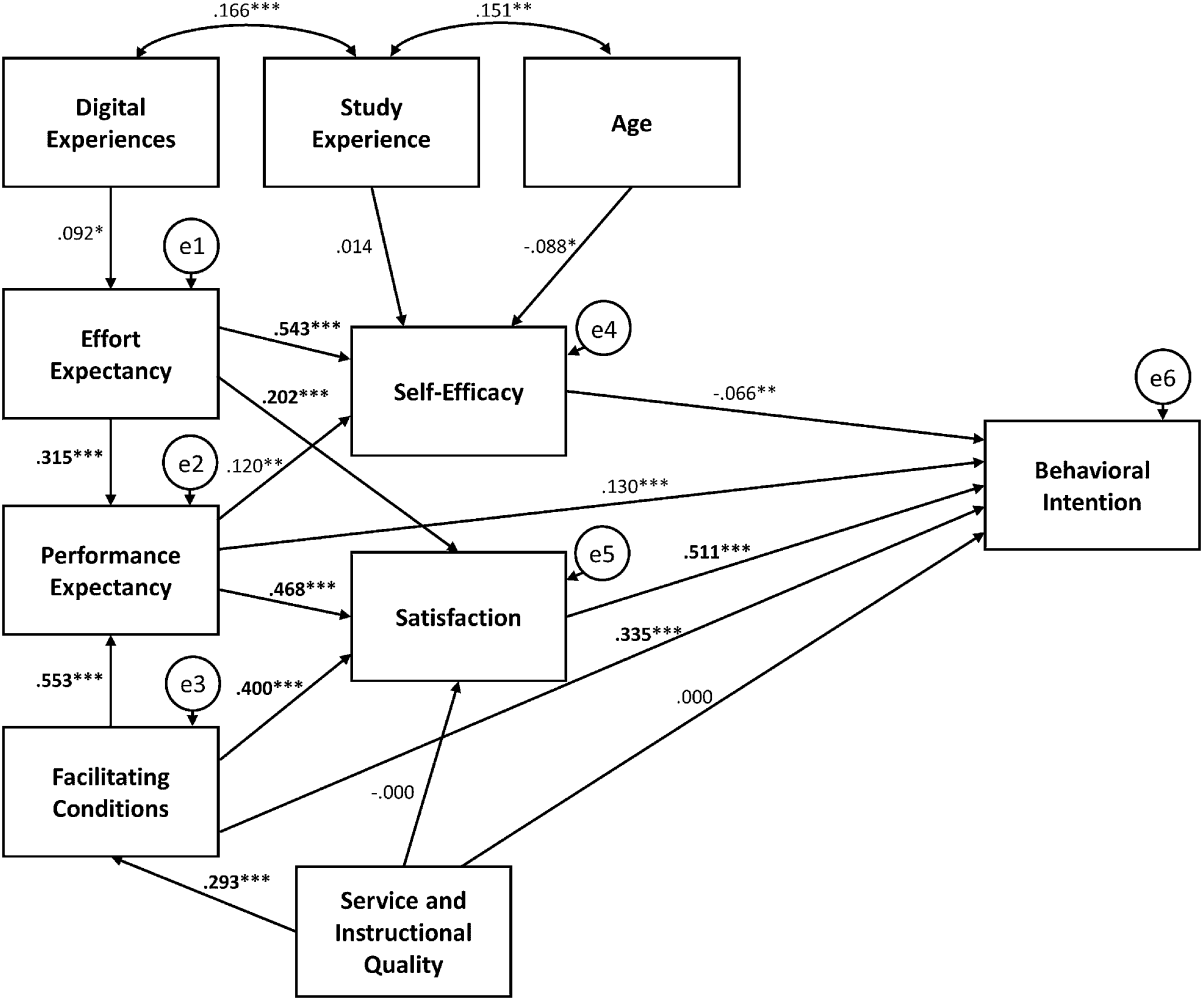
Baseline model of the path analysis. Significant effects are noted with *p < 0.05; **p < 0.01, and ***p < 0.001. e1–e6 denote errors due to unobserved exogenous variables

Based on the standardized regression weights, a baseline model of path analysis has been created to illustrate the effects of the latent variables on the criterion (see Fig. 4).

Model fit was estimated using five primary indices, as recommended by Hu and Bentler (1998) and others: the *normed fit index* (NFI) suggested by Bentler and Bonett (1980), Bollen’s (1989) *relative fit index* (RFI) and *incremental fit index* (IFI), and the more common TLI and CFI (Hu & Bentler, 1998). With NFI = 0.919, RFI = 0.860, IFI = 0.930, TLI = 0.879, and CFI = 0.930, the obtained baseline model can be considered an acceptable fit. However, the obtained RMSEA = 0.109 indicates only a moderate fit at best (Hoyle & Panther, 1995; Kenny et al., 2015).

On the whole, the baseline model agrees only partially with the proposed model shown in Fig. 2. Specifically, gender, attainment value, perceived enjoyment, self-management of learning, social influence, and ubiquity do not trigger in the obtained model, while 10 components of the proposed UTAUT model are enclosed in the baseline model shown in Fig. 4. In this model, the significant relatedness of *age*, *study experience*, and *digital experience* is intuitively plausible. The same holds true regarding the linkage of *service and instructional quality* and *facilitating conditions*. The model includes direct effects of the variable *facilitating conditions* on *performance expectancy*, *satisfaction*, and students’ *behavioral intention* (i.e., the criterion variable). Furthermore, *effort expectancy* has significant direct effects on *self-efficacy*, *performance expectancy*, and *satisfaction*, whereas *performance expectancy* directly affects *satisfaction*, *self-efficacy*, and, in particular, *behavioral intention*. Additionally, the obtained UTAUT model indicates several significant indirect effects of latent variables on the criterion variable. For example, *effort expectancy* has a significant effect *on behavioral intention* mediated by *self-efficacy*; notably, the variable *facilitating conditions* is not only a direct determinant of *behavioral intention* but also affects the dependent variable mediated by *performance expectancy* and *satisfaction*.

From a UTAUT perspective, these paths are definitely comprehensible. In particular, the interplay of the endogenous variables *effort expectancy*, *performance expectancy*, *self-efficacy*, and *satisfaction* can be considered theoretically sound from a psychological point of view. These findings suggest that the *facilitating conditions* (i.e., the availability of the resources needed to engage in digital learning) affect the students’ *performance expectancy*, which, in turn, has a strong effect on their *satisfaction* with digital learning. This is a strong determinant of *behavioral intention* to continue digital learning. Notably, the *facilitating conditions* also have a strong direct effect on the students’ *satisfaction* with digital learning. The observation that prior experience with digital learning affects the students’ *effort expectancy*, in turn influencing the extent of *self-efficacy*, is also intuitively plausible.

## Discussion

The present study was conducted at the end of the first wave of the COVID-19 pandemic, which led to a completely unforeseen replacement of face-to-face instruction with online courses. Several spontaneous studies on stakeholders’ acceptance of digital teaching showed general difficulties in adjusting to the suddenly changed requirements of higher education. Based on an analysis of 13 studies at German universities, Schumacher et al. (2021) summarize lecturers’ difficulties in switching to emergency remote teaching. These difficulties include an increased workload and the greater time required for student guidance, but the most important change involved a lack of proficiency and knowledge for the spontaneous and effectual design of digital learning environments. According to Bosse et al. (2020), presidents of German universities advanced the opinion that the shift to digital learning has been facilitated by lecturers’ engagement and successful crisis management as well as the available facilitating conditions and assistance. Specifically, the available technical equipment was considered to be supportive. Interestingly, all presidents assumed that students had critical (i.e., negative) attitudes toward digital higher education. This opinion appears to correspond to the findings of certain studies, which showed that students faced a number of problems related to the rapid shift to digital learning (e.g., Aristovnik et al., 2020; Händel et al., 2020; Traus et al., 2020).

However, in contrast to these studies, our results show that the majority of students were able to adjust to the requirements of pandemic-induced digital learning. Although our participants expressed some reservations and skepticism regarding performance expectancy and satisfaction, they positively evaluated their self-efficacy, attainment values, and capability of self-management of learning. Similar to the university presidents in the survey by Bosse et al. (2020), the students attached great importance to service and instructional quality and the facilitating conditions of the digital learning environment. The students regulated their effort expectancies depending on the degree of ease associated with the actual use of the materials provided in digital learning environments. On the whole, our results indicate that students possess the essential capability to cope with the challenges of emergency remote teaching and learning successfully.

Stimulated by the COVID-19 pandemic, our study aimed at investigating the fitness of the UTAUT model for meeting the demands of emergency remote studying. Since its advent, the UTAUT model has been used to investigate the optional use of information technology for school learning and higher education, but only a few studies have focused on mandatory digital learning (Carter et al., 2020). Furthermore, previous studies in the UTAUT field have focused on the acceptance and use of a specific technology, such as smartphones or tablets (e.g., Huan et al., 2015; Kang et al., 2015; Thomas et al., 2013), or learning management systems and webinars (e.g., Bervell & Umar, 2017; Bouznif, 2018; Khechine & Lakhal, 2018). In our study, digital technologies overall were of interest, whether virtual tutoring, video conferencing, or learning management systems. To that effect, the UTAUT-related questionnaire used in the present study incorporates 44 items, whereas studies focusing on a specific technology have incorporated far fewer items into their surveys. For instance, Chao’s (2019) questionnaire comprised 31 items, and Huan et al. (2015) included 32 items in their questionnaire. The different instrument sizes may result in diverging internal consistencies and factor loadings in CFA. In our study, several subscales of the UTAUT questionnaire (e.g., social influence, self-efficacy, and attainment value) indicated missing internal consistency between the items. This requires the continued development of the instrument to improve reliability. Despite this observation and the resulting recommendation for future work, the CFA clearly confirmed the construct validity of the UTAUT questionnaire inasmuch as the common fit indices met the conventional standards defined by Hu and Bentler (1998). They also correspond to the fit indices found in, for example, Huan et al. (2015), Dwivedi et al. (2019), and Thomas et al. (2013).

From a methodological point of view, a peculiarity of our study must be noted which may restrict its comparability with other studies. We used *polychoric correlations* for statistical analysis, whereas previous UTAUT studies commonly worked with Pearson coefficients. However, several studies have shown that polychoric correlations provide a more accurate assessment of the construct validity of surveys based on Likert scales (Asún et al., 2016; Holgado-Tello et al., 2010). In our study, using Pearson coefficients would have resulted in CFI = 0.900, TLI = 0.887, and RMSEA = 0.052. Therefore, polychoric correlations are beneficial in the case of Likert scales. Regardless, the validation of the UTAUT questionnaire with CFA provided factor loadings which correspond largely to those found in other studies (see Table 3).

**Table 3:** Comparison of factor loadings from several studies on UTAUT

UTAUT construct	This study	Huan et al. (2015)	Chao (2019)	Khechine and Lakhal (2018)	Romero-Rodríguez et al. (2020)
Behavioral intention	0.777–0.946	0.814–0.988	0.860–0.890		0.929–0.933
Perceived enjoyment	0.622–0.784	0.794–0.836	0.790–0.850		
Effort expectancy	0.632–0.909	0.763–0.839	0.730–0.800	0.856–0.890	0.792–0.856
Social influence	0.130–0.700	0.761 & 0.905		0.764–0.809	0.863–0.901
Facilitating conditions	0.289–0.917	0.840 & 0.866		0.927 & 0.927	0.688–0.820
Service and instructional quality	0.013–0.625	0.784–0.919			
Self-efficacy	0.208–0.578	0.770 & 0.704	0.850–0.880	0.769–0.851	
Performance expectancy	0.594–0.935	0.783–0.848	0.700–0.820	0.848–0.916	0.837–0.896
Satisfaction	0.671–0.919		0.700–0.820	0.730–0.885	
Self-management of learning	0.549–0.867	0.747–0.879			
Attainment value	0.003–0.846	0.921 & 0.927			
Ubiquity	0.583–0.737	0.784 & 0.754			

With the exception of the subscales *service and instructional quality* and *self-efficacy* (with low factor loadings and an inconsistency of items), the factor loadings found in the studies shown in Table 3 are collectively comparable. Notably, a particular lack of item consistency was reflected in our study with regard to *attainment value* and *social influence*, which was the only factor with negative loadings. Admittedly, this could be plausibly explained by the constraints imposed by mandatory digital learning, whereas in optional digital learning settings, *social influence* is reflected in the extent to which students are influenced by instructors or classmates to use digital technologies.

The specifics of mandatory digital learning also impact the results of the path analysis, which indicated that *social influence* and other subscales of the UTAUT questionnaire—such as perceived enjoyment, attainment value, and self-management of learning—did not trigger (i.e., they produced models with unacceptable fit indices). *Social influence* is considered a key factor in the original UTAUT model. In the studies by Khechine and Lakhal (2018), Salloum and Shaalan (2019), Tarhini et al. (2016), and Thomas et al. (2013), it proved to be a major determinant of behavioral intention to continue digital learning. In contrast, the studies by Huan et al. (2015), Bouznif (2018), and Romero-Rodríguez et al. (2020) showed that it exerted only negligible effects on *behavioral intention*. Regarding *perceived enjoyment*, *attainment value*, and *self-management of learning*, a basis for comparison is lacking because these components have not been investigated in many previous studies. The study by Huan et al. (2015) is an exception. In our study, the *facilitating conditions* did exert a major effect on *behavioral intention*. This is in contrast with the results reported by Huan et al. (2015) but conforms to the observations of Bervell and Umar (2017), Dulle and Minishi-Majanja (2011), Romero-Rodríguez et al. (2020), Tarhini et al. (2016), Thomas et al. (2013), and Dwivedi et al. (2019). According to these studies, the behavioral intention to continue digital learning is strongly affected by the facilitating conditions in digital learning environments. This indicates that in previous research, a parsimonious collection of four constructs—*performance expectancy*, *effort expectancy*, *social influence*, and *facilitating conditions—* has been suggested that may explain the behavioral intention to accept and use information technology for learning. The constructs *performance expectancy* and *effort expectancy* indicate individuals’ beliefs that digital learning may help to achieve academic success, provided that *effort expectancy* is satisfied by the learning environment. The *facilitating conditions* and *social influence* constructs may be viewed as contextual or organizational factors that influence individuals’ behavioral intention. However, in the case of *social influence*, the findings of the previous research are fairly inconsistent, whereas the effects of facilitating conditions appear unquestionable.

Notably, previous UTAUT studies seemed to be satisfied with investigating the direct effects of the core constructs on behavioral intention, as the indirect and interaction effects on behavioral intention to learn digitally have been measured in only a few studies. Dulle and Minishi-Majanja (2011), Khechine and Lakhal (2018), and Romero-Rodriguez et al. (2020) reported on the mediating effects of age, gender, and digital experience on the aforementioned core components of UTAUT. Additionally, our study indicated that students’ age, general study experience, and digital experience interacted with each other and exerted separate effects on effort expectancy and self-efficacy. Moreover, our results are incompatible with the interaction effect between facilitating conditions and digital experience described by Khechine and Lakhal (2018). This also holds true in terms of the (negatively loaded) interaction effects between social influence and digital experience and between social influence and voluntariness, which were also described by Khechine and Lakhal. Similarly, the results of the present study do not correspond to the interaction between effort expectancy and perceived risk or between performance expectancy and perceived risk reported by Chao (2019). Although Chao identified significant effects of satisfaction on behavioral intention that match the results of the present study, the explained effects of both self-efficacy and perceived enjoyment on satisfaction (and on effort expectancy and performance expectancy) are not in line with the observations of our study, according to which effort and performance expectancy affect self-efficacy. Furthermore, perceived enjoyment did not trigger at all. Consequently, the baseline model identified in this study does not conform to the baseline model of the path analysis described by Chao (2019). Essentially, this argument also applies to the work of Bervell and Umar (2017), which shows partial conformity with our study insofar as several paths in the baseline models are compatible. However, on the whole, the baseline model of the path analysis described by Bervell and Umar differs considerably from our baseline model depicted in Fig. 4.

## Limitations

As with all empirical research, it must be acknowledged that the present study has certain limitations. First, the participants were contacted via an announcement on their university’s learning management system and through email invitations from lecturers in large courses. Therefore, the study utilized a convenience sampling method, which limits the extent to which the results can be generalized. This asks for further investigation of the newly developed UTAUT- and data-based model of technology acceptance among students and their behavioral intention to use technology for learning (under the conditions of social distancing and emergency remote teaching). Second, the sample included only students. Future work could incorporate the perspective of lecturers who were charged with ensuring the continuation of higher education under pandemic-related constraints. Third, as implied above, the low reliability of certain subscales in the UTAUT questionnaire used in the current study indicate the need for a further development of the measurement instrument. Finally, the findings cannot be readily generalized to higher education and digital learning overall, as the data were collected during the pandemic and are related to emergency remote teaching.

## Conclusion

It is fair to conclude that despite its limitations, the present study adds to the research body on the intrapersonal and external factors affecting university students’ technology acceptance and their behavioral intention to use technology for learning in phases of social distancing and emergency remote teaching. Additionally, the study contributes to the validation of UTAUT, which has been proven to be a solid theoretical framework for applied research on digital learning in higher education. Our findings not only highlight the variables that are important in shaping students’ behavioral intention but also indicate certain underlying dependencies (i.e., indirect effects). Considering these factors and their relationship (see Fig. 4) can be useful in designing and implementing remote teaching scenarios and digital learning environments.

## Data Availability

The datasets used and/or analyzed during the current study are available from the corresponding author on reasonable request.
